# Comparative genomics of host-specialized populations of *Corynespora cassiicola* causing target spot epidemics in the southeastern United States

**DOI:** 10.3389/ffunb.2022.910232

**Published:** 2022-07-22

**Authors:** Leilani S. Dacones, Robert C. Kemerait, Marin T. Brewer

**Affiliations:** ^1^ Department of Plant Pathology, University of Georgia, Athens, GA, United States; ^2^ Department of Plant Pathology, University of Georgia, Tifton, GA, United States

**Keywords:** mating-type, *MAT1*, necrotrophic effector, cassiicolin, T-toxin

## Abstract

Numerous plant-pathogenic fungi secrete necrotrophic effectors (syn. host-selective toxins) that are important determinants of pathogenicity and virulence in species that have a necrotrophic lifestyle. *Corynespora cassiicola* is a necrotrophic fungus causing emerging target spot epidemics in the southeastern United States (US). Previous studies revealed that populations of *C. cassiicola* from cotton, soybean, and tomato are clonal, host specialized and genetically distinct. Additionally, cassiicolin – the necrotrophic effector identified in some *C. cassiicola* isolates – is an important toxin for virulence on rubber. It is encoded by seven *Cas* gene variants. Our goal was to conduct comparative genomic analyses to identify variation among putative necrotrophic effector genes and to determine if lack of one of the mating-types explained clonal populations in *C. cassiicola* causing outbreaks in the southeastern US and the apparent absence of sexual reproduction worldwide. A total of 12 C*. cassiicola* genomes, with four each from isolates from tomato, soybean, and cotton, were sequenced using an Illumina Next Seq platform. Each genome was assembled *de novo*, compared with the reference genome from rubber, and searched for known *Cas*, and other gene clusters with homologs of secondary metabolites. *Cas2* and/or *Cas6* were present in isolates from soybean in the southeastern US, whereas *Cas1 and Cas2* were present in isolates from cotton in the southeastern US. In addition, several toxin genes, including the T-toxin biosynthetic genes were present in all *C. cassiicola* from cotton, soybean, and tomato. The mating-type locus was identified in all of the sequenced genomes, with the *MAT1-1* idiomorph present in all cotton isolates and the rubber isolate, whereas the *MAT1-2* idiomorph was present in all soybean isolates. We developed a PCR-based marker for mating-type in *C. cassiicola*. Both mating types were present in isolates from tomato. Thus, *C. cassiicola* has both mating-types necessary for sexual reproduction, but the absence of both mating-types within soybean and cotton populations could explain clonality in these populations. Variation in necrotrophic effectors may underlie host specialization and disease emergence of target spot on cotton, soybean, and tomato in the southeastern US.

## Introduction

Evolutionary processes play a vital role in shaping plant disease epidemics and populations of their causal pathogens over time and space (Milgroom and Peever, 2003). In agricultural environments extensive monoculture combined with disease management practices that result in strong selective pressures, especially single-site fungicides and host plant resistance mediated by a single gene, can drive evolutionary arms races over relatively short timescales. These selective pressures contribute to changes in virulence and major shifts in the predominant pathogen populations interacting with the hosts and causing disease outbreaks. An example of a major shift is the rapid emergence in the 1970’s of *Bipolaris maydis* (syn. *Cochliobolus heterostrophus*) race T that caused severe epidemics of Southern Corn Leaf Blight (SCLB). Race T produces T-toxin (Kono et al., 1980), which was lethal to the widely-planted Texas cytoplasmic male sterile maize (T-cms) (Dewey et al., 1988; Levings, 1990). T-toxin is a host-selective toxin (syn. necrotrophic effector) produced by *B. maydis* race T (Kono and Daly, 1979) that is absent in *B. maydis* race O, which was not very virulent and had been the predominant race prior to the SCLB epidemics. The maize mitochondrial protein, *Urf13* (Levings, 1993), conferring male sterility also confers sensitivity to T-toxin, thereby resulting in elevated virulence of race T and widespread epidemics of SCLB in the 1970’s. In addition to increasing virulence, host selective toxins also contribute to the evolution of host specificity.

Plant pathogens are often adapted to different host species, or even more specifically to different host cultivars (Burdon and Silk, 1997). The initial cultivar or host jump may lead to the breakdown of host resistance and emergence of a new disease. Over time population genetic subdivision resulting from host specialization results in genetic differences and allows pathogens to maintain population diversity and subsequently help to evade extinction (Fournier and Giraud, 2007). An understanding genetic differentiation by host aids in implementing proper disease management. Host specialization is particularly common for fungal pathogens with biotrophic lifestyles, such as rusts and powdery mildews, that have intimate associations with their host (Duplessis et al., 2011; Ohm et al., 2012). For necrotrophic plant pathogens, host specialization often involves mechanisms such as the production of host-selective toxins (Friesen et al., 2008; Friesen and Faris, 2012), or acquisition of conditionally dispensable chromosomes (also known as supernumerary, accessory, or lineage-specific chromosomes) (Hatta et al., 2002; Ma et al., 2010; Vlaardingerbroek et al., 2016) that carry genes for pathogenicity or virulence that can be moved *via* horizontal gene transfer (HGT) (Soanes and Richards, 2014). *Pyrenophora tritici-repentis* is a necrotroph that causes tan spot of wheat. It is virulent on wheat due to the production of the necrotrophic effector ToxA that mediates host-specific interactions (Ciuffetti et al., 1997). The increase in virulence was attributed to the acquisition of ToxA *via* HGT (Friesen et al., 2006) from another wheat pathogen, *Stagonospora nodorum*, which also produces ToxA. Another example where necrotrophic effectors are involved in virulence and host specificity is in *Alternaria alternata*, which has a wide host range (Gilchrist and Grogan, 1976); however, host specificity of pathotypes conferred by diverse necrotrophic effectors results in at least 11 different diseases of unique host plants (Tsuge et al., 2013).

*Corynespora cassiicola* is a ubiquitous saprotrophic and necrotrophic fungus commonly found in tropical and subtropical areas (Dixon et al., 2009). It has a wide host range across plant species (Farr and Rossman, 2017), but may be best known for causing Corynespora leaf fall of rubber (*Hevea brasiliensis*), a devastating disease resulting in severe economic losses in Asia and Africa (Chee, 1990). Recently, *C. cassiicola* has been causing emerging target spot epidemics in the southeastern United States (US) on cotton (*Gossypium hirsutum*) (Campbell et al., 2012; Edmisten, 2012; Fulmer et al., 2012; Mehl and Phipps, 2013; Price et al., 2015; Butler et al., 2016), soybean (*Glycine max*) (Koenning et al., 2006; Bennett, 2016; Faske, 2016; Edwards Molina et al., 2022), and tomato (*Solanum lycopersicon*) (Schlub et al., 2009). Phylogenetic and population genetic analyses of *C. cassiicola* from cotton, soybean, and tomato in the southeastern US showed three genetically distinct populations that clustered based on the host of origin (Sumabat et al., 2018a; Sumabat et al., 2018b). In addition, isolates were shown to be most aggressive when inoculated on the same host as the host of origin providing evidence for host specialization. The underlying genetic basis for host specialization in these populations is unknown.

As a necrotroph *C. cassiicola* kills plant tissue, which is most often the foliage and sometimes fruits. Characteristic symptoms include necrotic lesions forming a target-like appearance with concentric rings surrounded by a yellow margin. Often, these symptoms are followed by leaf drop or premature defoliation of both mature and immature leaves (Fulmer et al., 2012). Severe infections can lead to massive defoliation and subsequent death of infected plants (de Lamotte et al., 2007). The symptoms of target spot are characteristic of toxin involvement. Cassiicolin – a phytotoxic protein – is a necrotrophic effector in *C. cassiicola* isolates from rubber and determined to play a role in pathogenicity and virulence (Breton et al., 2000; de Lamotte et al., 2007). The precursor of cassiicolin is encoded by the gene *Cas1*, which is expressed by *C. cassiicola* in the early stages of infection of rubber (Déon et al., 2012). Seven variants of cassiicolin-encoding genes (*Cas1* to *Cas7*) have been identified among isolates from different hosts and diverse geographic regions (Déon et al., 2014; Lopez et al., 2018). The *Cas* gene variants encode different isoforms of the cassiicolin toxin. The variants are located in different regions of *C. cassiicola* genomes and when more than one variant was detected in a single genome they were not clustered (Lopez et al., 2018). Only 47% of the characterized isolates had *Cas* genes encoding for cassiicolin; however, isolates without *Cas* genes were still virulent, including some to rubber, showing that factors other than cassiicolin are involved in virulence and host specialization (Déon et al., 2014). A secreted toxin in culture filtrate was determined to play a role in pathogenicity of *C. cassiicola* to tomato but was not further characterized (Onesirosan et al., 1975). The role of *Cas* variants in pathogenicity and virulence of *C. cassiicola* populations causing epidemics in the southeastern US is unknown.

In fungal populations causing emerging plant diseases sexual reproduction is often absent and reproduction is strictly clonal. Sexual reproductive structures have not been observed in *C. cassiicola* and it is considered strictly clonal and assumed to only reproduce asexually (Schoch et al., 2009). All phylogenetic and population genetic studies to date, including those on the host-specialized populations of *C. cassiicola* causing emerging target spot epidemics in the southeastern US, indicate that *C. cassiicola* is clonal and lacks sexual reproduction (Silva et al., 2002; Dixon et al., 2009; Déon et al., 2014; Sumabat et al., 2018a; Sumabat et al., 2018b). Sumabat et al. (2018a) showed evidence of recombination among the *C. cassiicola* lineages from different hosts in the southeastern US. However, these recombination events did not likely occur recently (Sumabat et al., 2018b) or could be due to homoplasy since the reticulations were detected deep among the lineages. Aside from recombination, another indirect molecular method to infer sexual reproduction in fungi involves detection of compatible mating-types within the species or population of interest (Fraser and Heitman, 2003). The mating-type locus, *MAT1*, is present in both sexually and asexually reproducing members of Ascomycota (Debuchy and Turgeon, 2006). In heterothallic Ascomycota, *MAT1* has two variants, or idiomorphs, which together contain the genes necessary for sexual reproduction to occur; individuals must be different idiomorphs at *MAT1* to be sexually compatible. In homothallic Ascomycota, all of the genes for mating are present in a single individual. Therefore, the structure of *MAT1* in a species and composition of idiomorphs in a population provide valuable information on the mating system of that fungal species or population. Until now the *MAT1* locus in *C. cassiicola* had not been described, so it was unknown if the species is homothallic or heterothallic, or if populations are not able to reproduce sexually since they are lacking one of the mating-type idiomorphs.

The objectives of this study were to conduct comparative genomic analysis of host-specialized populations of *C. cassiicola* to: 1) identify variation among putative necrotrophic effector genes, including *Cas* variants and secondary metabolites, and 2) to identify and characterize *MAT1* in *C. cassiicola* and variation in mating-types among populations causing outbreaks in the southeastern US. Knowledge on the diversity of necrotrophic effector genes and the reproductive biology is critical in understanding the genetic basis of host specialization and disease emergence of target spot of cotton, soybean, and tomato in the southeastern US.

## Materials and methods

### DNA extraction and genome sequencing

Twelve *C. cassiicola* isolates from the three host-specialized populations in the southeastern US – cotton, soybean, and tomato – were selected for whole genome sequencing (**Table 1**). High quality genomic DNA was extracted using the cetyl trimethylammonium bromide (CTAB) method (Fulton et al., 1995) adapted from the 1000 Fungal Genome Project of Joint Genome Institute – Department of Energy (JGI-DOE, 1000.fungalgenomes.org). Briefly, isolates were grown on quarter-strength potato dextrose agar (qPDA) overlaid with sterile cellophane and incubated at 25°C for 7 days in the dark. Mycelium of each isolate was scraped from the cellophane with a spatula and ground in liquid nitrogen. Approximately 500 mg finely-ground mycelium was mixed with 17.5 ml CTAB lysis buffer, which consisted of: 6.5 ml Buffer A (0.35 M sorbitol; 0.1 M Tris-HCl, pH 9; and 5 mM EDTA, pH 8), 6.5 ml Buffer B (0.2 M Tris-HCl, pH 9; 50 mM EDTA, pH 8; 2 M NaCl; and 2% CTAB), 2.6 ml of Buffer C (5% Sarkosyl), 1.75 ml PVP (0.1%), and 1.25 μl Proteinase K. The mixture was shaken with two 5-mm glass beads (VWR Soda Lime, Radnor, PA, USA) at 1750 RPM for 2 min followed by 1 min using a 2010 Geno/Grinder (SPEX SamplePrep, Metuchen, NJ, USA). Next, 5.75 ml of 5 M potassium acetate was added to the tube then it was inverted 10 times. The mixture was incubated on ice for 30 min then centrifuged for 20 min at 14, 000 g. The supernatant was added to one volume of chloroform:isoamylalcohol (v/v 24:1) and subsequently centrifuged at 14, 000 g for 10 min. The resulting supernatant was mixed with 100 μl Rnase A (10 mg/ml) and incubated at 37°C for 2 hr. Isopropanol at equal volume and sodium acetate at 1/10 volume were then added, incubated at 25°C for 5 min, and centrifuged at 14, 000 g for 30 min. The supernatant was discarded, and the resulting pellet was rinsed twice with 70% ethanol and air-dried overnight. The DNA pellet was eluted with 500 μl deionized H_2_O. Genomic DNA was submitted to the Georgia Genomics and Bioinformatics Core (Athens, GA, USA) for library preparation of each isolate and Illumina sequencing using NextSeq platform based on a paired-end 150-bp (PE150) protocol. The genome of *C. cassiicola* isolate CCP from rubber (Lopez et al., 2018) was downloaded from NCBI (assembly ID: GCA_003016335.1, BioProject accession: PRJNA234811) for further analyses.

**Table 1:** Origin and basic metrics of the assembled *Corynespora cassiicola* genomes.

Host	Location	Isolate	Total length (bp)	N50 (bp)^1^	L50^2^	BUSCO^3^	NCBI Accession Nos.
Cotton (G*ossypium hirsutum*)	Macon Co., AL	CAL-4	47 452 810	105 987	137	285 (98.2%)	VICL00000000
	Duval Co., FL	FlM4	49 060 639	101 672	144	284 (97.9%)	JAMKBH000000000
	Mitchell Co., GA	CM13	53 858 206	34 439	431	284 (97.9%)	JAMQYD000000000
	Suffolk, VA	CVa5	45 300 321	85 044	164	284 (97.9%)	VICK00000000
Soybean (*Glycine max*)	Poinsett Co., AR	SAR-9	47 594 148	119 589	116	286 (98.6%)	JAMKBG000000000
	Marion Co., GA	SMR2	48 431 661	106 892	133	284 (97.9%)	JAMKBF000000000
	Tift Co., GA	Ssta1	44 988 225	57 801	221	285 (98.2%)	JAMKBE000000000
	Madison Co., TN	STNa-1	47 162 684	127 016	116	286 (98.6%)	JAMQYC000000000
Tomato (*Solanum lycopersicum*)	Hillsborough Co., FL	1343	56 850 383	97 921	159	285 (98.2%)	JAMQYB000000000
	Hillsborough Co., FL	1551	50 077 706	79 145	172	286 (98.6%)	JAMKBD000000000
	Cairo, GA	TCl3	45 966 631	48 911	270	285 (98.2%)	VICJ00000000
	Cairo, GA	TCf2^3^	50 844 802	77 901	180	286 (98.6%)	JAMKBC000000000

^1^Sequence length past 50% of the total assembly.

^2^Number of sequences past 50% of the total assembly.

^3^Isolate collected from tomato fruit.

### Genome assembly and quality metrics

Illumina short reads from the replicated runs were concatenated to their corresponding read direction (all forward reads together; all reverse reads together) for each of the sequenced isolates. Quality assessment of the raw reads was conducted using FastQC v.0.11.4 (Andrews, 2010) on the Georgia Advanced Computing Resource Center (GACRC) Linux cluster. Trimming was performed using TrimGalore v.0.3.7 (Krueger, 2015) to remove adapter, and other short and low-quality sequences. Filtering parameters were set based on the initial evaluation of the raw reads with FastQC. Trimmed paired-end read files for each isolate were assembled *de novo* using SPAdes v.3.11.0 (Bankevich et al., 2012). Optimal *k*-mer values were based on the iterative feature of SPAdes that selects those that exhibit the best quality metrics from multiple *k*-mer values. The resulting genome assemblies were assessed using QUAST v.4.5 (Gurevich et al., 2013) and BUSCO v.3.0.2 (Kriventseva et al., 2015) with the conserved fungal dataset “Fungi odb9” that contains 290 genes. The 12 draft genomes (**Table 1**) and raw reads were deposited at NCBI under BioProject number PRJNA549429

### *In silico* identification and phylogenetic analyses of cassiicolin-encoding genes

Each of the 12 C*. cassiicola* assembled draft genomes, as well as the reference genome from the rubber isolate CCP, was searched for homologs of the *Cas* genes (Déon et al., 2014) using NCBI BLAST+ (Altschul et al., 1990) with the following nucleotide sequences of *C. cassiicola*: *Cas1* from isolate CCP from rubber (JF915148), *Cas2* from isolate ATI17 from cotton (JF915159), *Cas3* from isolate E70 from rubber (JF915169), *Cas4* from isolate E79 from rubber (JF915171), *Cas5* from isolate SS1 from rubber (JF915173), *Cas6* from isolate ATI17 from cotton (JF915182), and *Cas7* from isolate IA from cucumber (MF564202).

Nucleotide sequences of *Cas* genes identified by BLAST+ in the 12 C*. cassiicola* draft genomes and the reference genome, as well as sequences representative of the 6 *Cas* variants, were visually edited and aligned in Geneious v.7 (Biomatters) using ClustalW (Thompson et al., 2002). Phylogenetic analyses were performed using Maximum Likelihood (ML) in MEGA5 (Tamura et al., 2011) and Bayesian Inferences (BI) in MrBayes (Ronquist and Huelsenbeck, 2003). An evolutionary model of nucleotide substitution was determined based on goodness-of-fit in MEGA5. The Tamura three-parameter model of evolution assuming a γ distribution with invariant sites was identified as the most appropriate. Support for each node was determined by 500 bootstrap replicates for ML whereas four incrementally heated Markov chains were run, and samples were taken every 100 generations for 5,000,000 generations for BI. To compare differences in coding regions among the variants, the predicted amino acid sequences of the identified cassiicolin-encoding genes were aligned in Geneious v.7 using ClustalW.

### Identification of additional putative necrotrophic effectors and mating-type genes

To identify putative necrotrophic effector genes, each of the *C. cassiicola* draft genomes and the reference genome were scanned for biosynthetic loci of known secondary metabolite compound classes in fungi using the webserver tool of the antiSMASH pipeline (Blin et al., 2011). Gene prediction was performed through GlimmerHMM (Pertea et al., 2004) with the FASTA file of the input eukaryotic data. The amino acid sequence translations of all protein-encoding genes were mined with profile Hidden Markov Models (pHMM) using the HMMer3 tool (Eddy, 2009). The models are based on multiple sequence alignments of previously described protein signatures or protein domains. Subsequent BLAST searches were performed on the 12 C*. cassiicola* draft genomes and the reference genome to query individual biosynthetic genes of the secondary metabolite initially identified by the antiSMASH pipeline.

To identify the mating-type locus in *C. cassiicola* each of the draft genomes and the reference genome were scanned for *MAT1* by BLAST searches. The mating-type genes *MAT1-1-1* (AAB82945) and *MAT1-2-1* (AAB84004) as well as the flanking genes *GTPase activating protein* (*gap1*, AAB82943), unknown open reading frame (*orf1*, AAB82944), and *ß-glucosidase* (*bgl1*, AAB82946) from *B. maydis* were used as queries.

### Development of a multiplex PCR-based assay for mating-type in *C. cassiicola*

PCR primers (**Table 2**) were designed to amplify sequences of the *MAT1-1* and *MAT1-2* idiomorphs of *C. cassiicola* isolates in a multiplex reaction. The expected fragment size for *MAT1-1* was 1494 bp, whereas for *MAT1-2* was 998 bp. The PCR was initially tested on the 12 C*. cassiicola* isolates sequenced by Illumina since their mating-type idiomorphs were known based on BLAST searches. Multiplex PCR amplification was performed in a 12.5 μl reaction containing the following: 1.25 μl of 10× PCR buffer (Takara Bio Inc., San Jose, CA, USA), 1.25 μl dNTPs (2.5 mM each), 0.56 μl each of the 10 μM primers CCMAT1-1F2, CCMAT1-1R2, CCMAT1-2F4, and CCMAT1-2R4, 0.75 U ExTaq (Takara Bio Inc.), and 1 μl (20-300 ng) DNA template. Thermal cycling conditions had an initial denaturation for 5 min at 94°C followed by; 28 cycles of 30 s at 94°C, 30 s at 52°C, and 30 s at 72°C; and a final elongation of 2 min at 72°C. Confirmation of PCR products was performed by electrophoresis on a 1% (w/v) agarose gel with 1×TBE buffer. PCR products were cleaned with ExoSAP-IT PCR product cleanup reagent (Affymetrix, Santa Clara, CA, USA). Representative DNA fragments were sent for Sanger sequencing (Eurofins Scientific, Louisville, KY, USA). All PCR products were sequenced in both directions using the same primers used for PCR.

**Table 2:** Primers for multiplex PCR-based mating-type (*MAT1*) marker in *C. cassiicola*.

Primer name	Sequence (5’-3’)	Target amplicon size (bp)
CCMAT1-1F2	TGTGCTGAG TTGAGT TAGCGT	1494
CCMAT1-1R2	AGGTGATGTTGATCAATAGCCGT	
CCMAT1-2F4	TCCATCACACACTCTTTGCAC	998
CCMAT1-2R4	CTGGGTCAAGAATGCCGATA	

The multiplex PCR-based assay for mating type was conducted on 58 additional *C. cassiicola* isolates causing recent epidemics in the southeastern U.S., including 25 from cotton, 20 from soybean, and 13 from tomato to identify their mating type. These isolates came from different fields across states (Georgia, Florida, Alabama, Mississippi, Tennesee, Louisiana, Virginia) where epidemics were severe, and were sampled over multiple years (Sumabat et al., 2018a; Sumabat et al., 2018b).

## Results

### Genome statistics

Twelve *C. cassiicola* isolates were sequenced and draft genomes were assembled (**Table 1**). The optimal *k*-mer value determined in SPAdes was *k* = 77 for all genomes. Assembled genome size ranged from 45.0 to 56.9 Mbp. The N50 ranged from 34 to 127 Kbp with a mean of 87 Kbp. The L50 ranged from 116 to 431 with a mean of 187 contiguous sequences. Genome completeness values measured in BUSCO ranged from 97.9% to 98.6% complete for the 290 conserved reference genes in the dataset “Fungi odb9”.

### *In silico* identification of cassiicolin-encoding genes and phylogenetic analysis of *cas* variants

**Figure 1:**
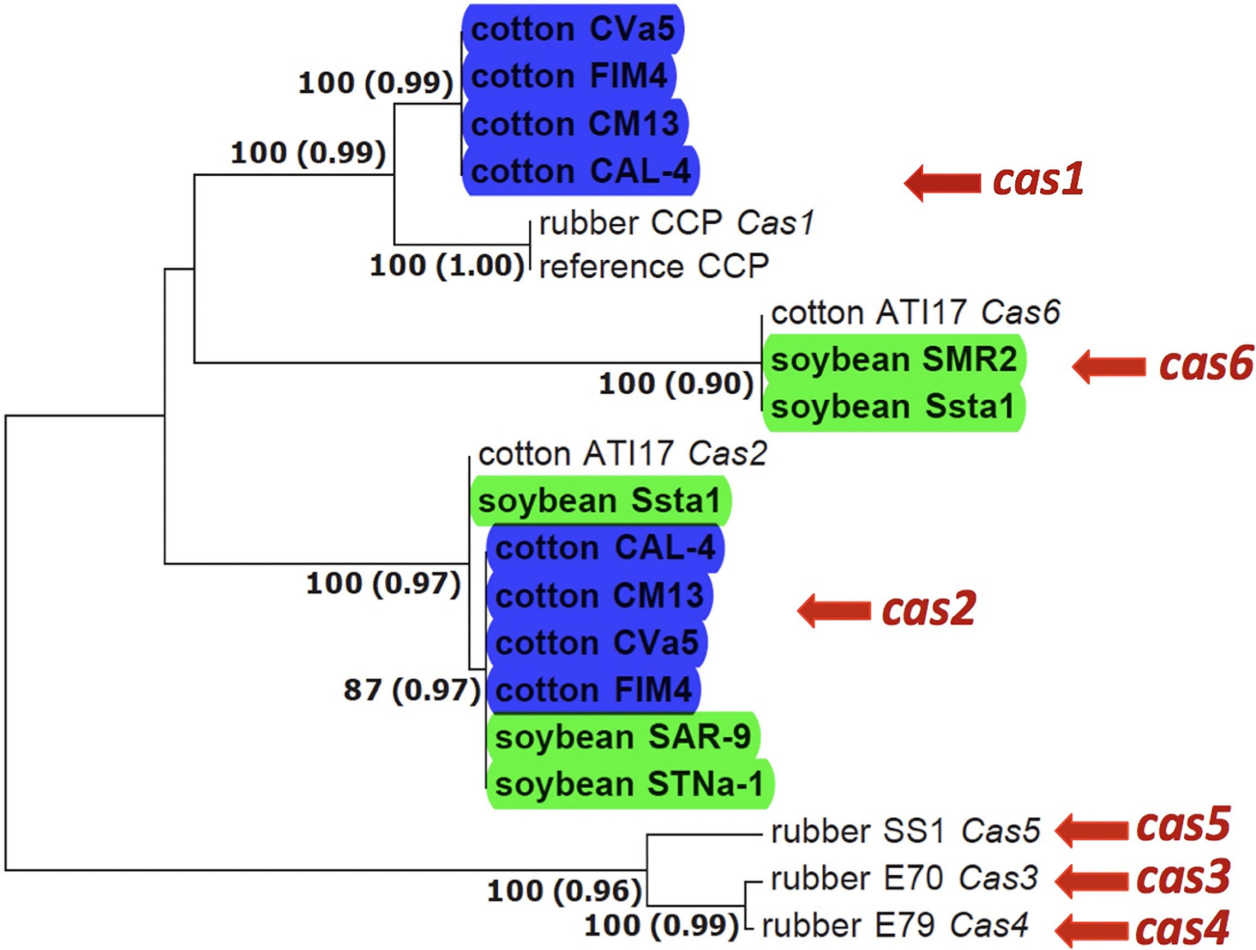
Phylogenetic tree of the cassiicolin-encoding genes in *Corynespora cassiicola* from different hosts and geographic regions based on Maximum Likelihood (ML) and Bayesian Inference (BI). *C. cassiicola* genomes sequenced and used in this study are in bold and highlighted blue (cotton) and green (soybean). ML bootstrap values > 70% are shown before the parenthesis for each of the supported branches. Posterior probabilities > 90% are shown in parenthesis. Original hosts and isolate names are indicated for all isolates. *Cas7*, which is divergent from the other *Cas* variants, especially for nucleotide sequences, and not found in any of the genomes from this study (Lopez et al., 2018), is not included.

**Figure 2:**
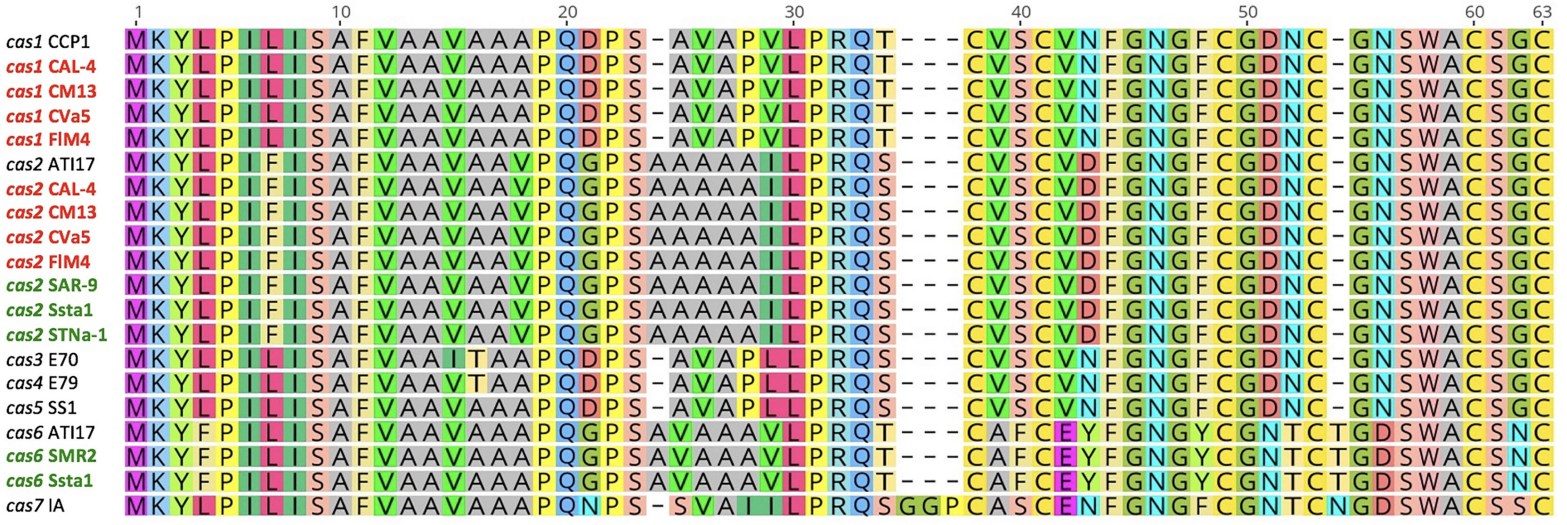
Amino acid sequence alignment of the cassiicolin precursor proteins of the 7 known *Cas* variants in *Corynespora cassiicola*. *Cas* designation and isolate name are shown before each corresponding sequence. Isolates that we collected and sequenced for this study are indicated in bold. The sequences with names in red are from cotton isolates and the sequences with names in green are from soybean isolates.

BLAST analysis using nucleotide sequence queries revealed homologs of cassiicolin-encoding genes in the assembled genomes of *C. cassiicola* from cotton and soybean. The genes were identified to *Cas* variant based on how they clustered with known *Cas* variants in the gene tree (**Figure 1**) and their amino acid identity with known *Cas* variants (**Figure 2**). We found more than one *Cas* variant in some of the *C. cassiicola* isolates (**Supplemental Table S1**). All four sequenced *C. cassiicola* isolates from cotton have both *Cas1* and *Cas2*. Although the nucleotide sequences of the *Cas1* precursor genes from cotton isolates from the southeastern US vary by more than 20 nucleotides from the reference *Cas1* variants (**Figure 1**), the translated amino acid sequences are identical (**Figure 2**). One soybean isolate (Ssta1) has both *Cas2* and *Cas6*. The other isolates from soybean, SAR-9 and STNa-1 have only *Cas2*, whereas isolate SMR2 has only *Cas6*. When more than one *Cas* variant was detected within the genome of a single isolate, the variants were found on different contigs (**Supplemental Table S1**). No *Cas* variants were detected in any of the *C. cassiicola* assembled genomes from tomato.

### *In silico* identification of additional putative necrotrophic effectors and mating-type genes

**Figure 3:**
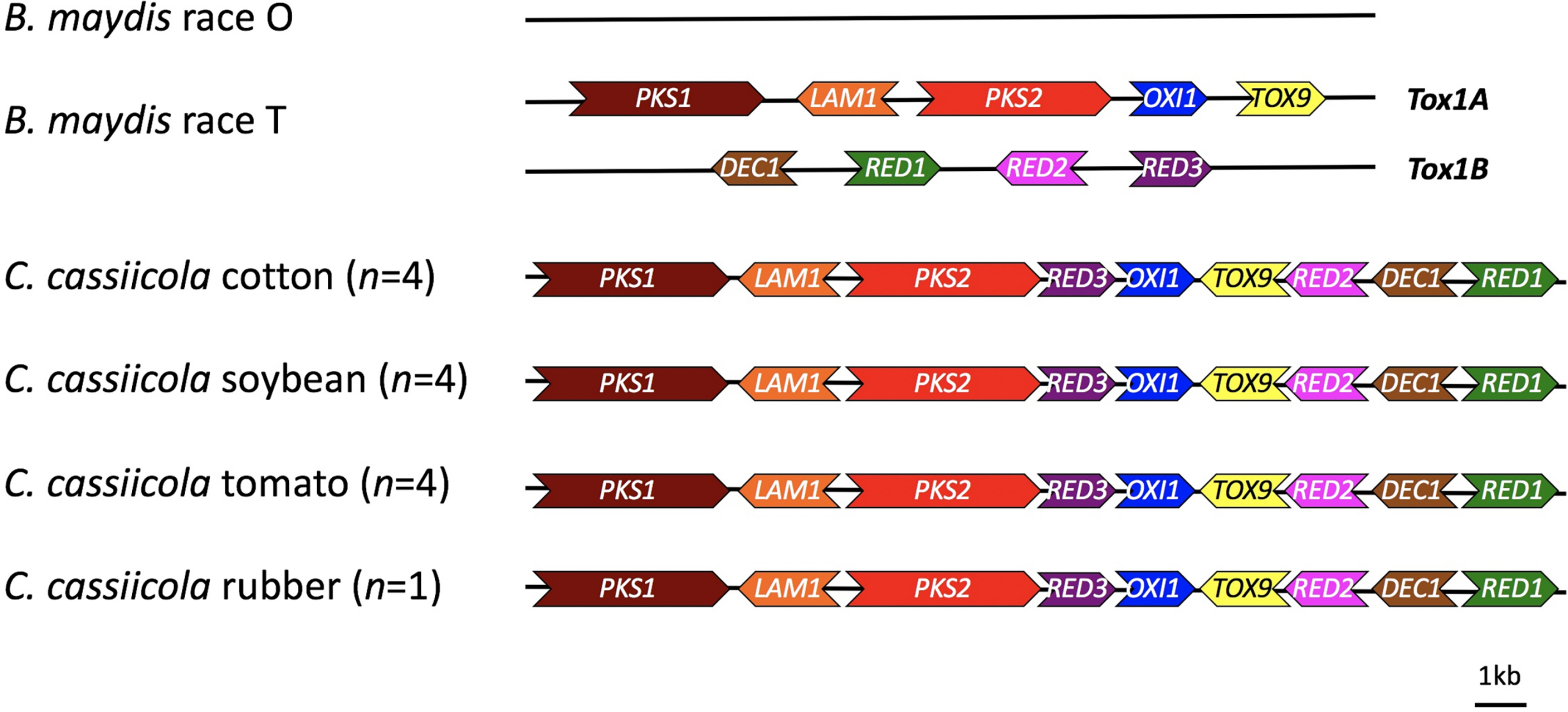
Schematic diagram of the structure of T-toxin biosynthetic genes identified in *Corynespora cassiicola* isolates sampled and sequenced for this study. The genes *pks1* (AAB08104.3), *lam1* (ACP43390.1), *pks2* (ABB76806.1), *oxi1* (ADB23430.1), *tox9* (ADB23431.1), *dec1* (AAM88291.1), *red1* (AAM88292.1), *red2* (ACP34152.1), and *red3* (ACP34153.1) from *Bipolaris maydis* race T were used as references for comparison.

A web-server tool of the antiSMASH pipeline was used to identify additional putative necrotrophic effectors or secondary metabolite gene clusters in *C. cassiicola*. Orthologs for genes needed for synthesis of T-toxin were detected in all of the assembled genomes of *C. cassiicola* from cotton, soybean, tomato, and rubber with high similarity to the biosynthetic genes of T-toxin in *B. maydis* (**Figure 3**). These gene clusters were located on a single contig in each of the *C. cassiicola* genomes. Other putative toxin gene clusters detected *in silico* in the *C. cassiicola* genomes showed similarity to gene clusters for brefeldin, ectoine, terrein, aspirochlorine, zearalenone, and acetylaranotin (**Table 3**). Some of these secondary metabolite gene clusters, including the one for zearalenone, had two similar gene clusters in all *C. cassiicola* genomes. A cluster with genes similar to the brefeldin cluster was found in all cotton and soybean isolates however, absent in all tomato isolates and the rubber isolate. A gene cluster with genes similar to those in the ectoine biosynthetic cluster was uniquely identified in one tomato isolate, whereas a cluster with genes similar to the terrein cluster was only found in two cotton isolates. A cluster similar to the aspirochlorine biosynthetic cluster was detected in all soybean and two tomato isolates, whereas on cotton isolates this cluster was lacking. A gene cluster with similarity to acetylaranotin was detected in all cotton isolates and two tomato isolates. Aside from the T-toxin cluster, none of these identified clusters contained all of the genes necessary for synthesis of the known secondary metabolite.

**Table 3:** Gene clusters identified in the *Corynespora cassiicola* genomes containing genes with similarity to toxin gene clusters.

Isolate (host)	Brefeldin	Ectoine	Terrein	Aspirochlorine	Zearalenone	Acetylaranotin
CAL-4 (cotton)	(+)^1^	(-)	(-)	(-)	(++)	(+)
FlM4 (cotton)	(+)	(-)	(+)	(-)	(++)	(+)
CM13 (cotton)	(+)	(-)	(-)	(-)	(++)	(+)
CVa5 (cotton)	(++)	(-)	(+)	(-)	(++)	(+)
SAR-9 (soybean)	(+)	(-)	(-)	(+)	(++)	(-)
SMR2 (soybean)	(+)	(-)	(-)	(+)	(++)	(-)
Ssta1 (soybean)	(+)	(-)	(-)	(+)	(++)	(-)
STNa-1 (soybean)	(+)	(-)	(-)	(+)	(++)	(-)
1343 (tomato)	(-)	(+)	(-)	(+)	(++)	(-)
1551 (tomato)	(-)	(-)	(-)	(-)	(++)	(+)
TCl3 (tomato)	(-)	(-)	(-)	(-)	(++)	(+)
TCf2 (tomato)	(-)	(-)	(-)	(+)	(++)	(-)
CCP (rubber)	(-)	(-)	(-)	(-)	(++)	(-)

^1^(+/-) indicates presence/absence of a cluster with similar genes; number of (+) indicates number of clusters with similarity identified.

**Figure 4:**
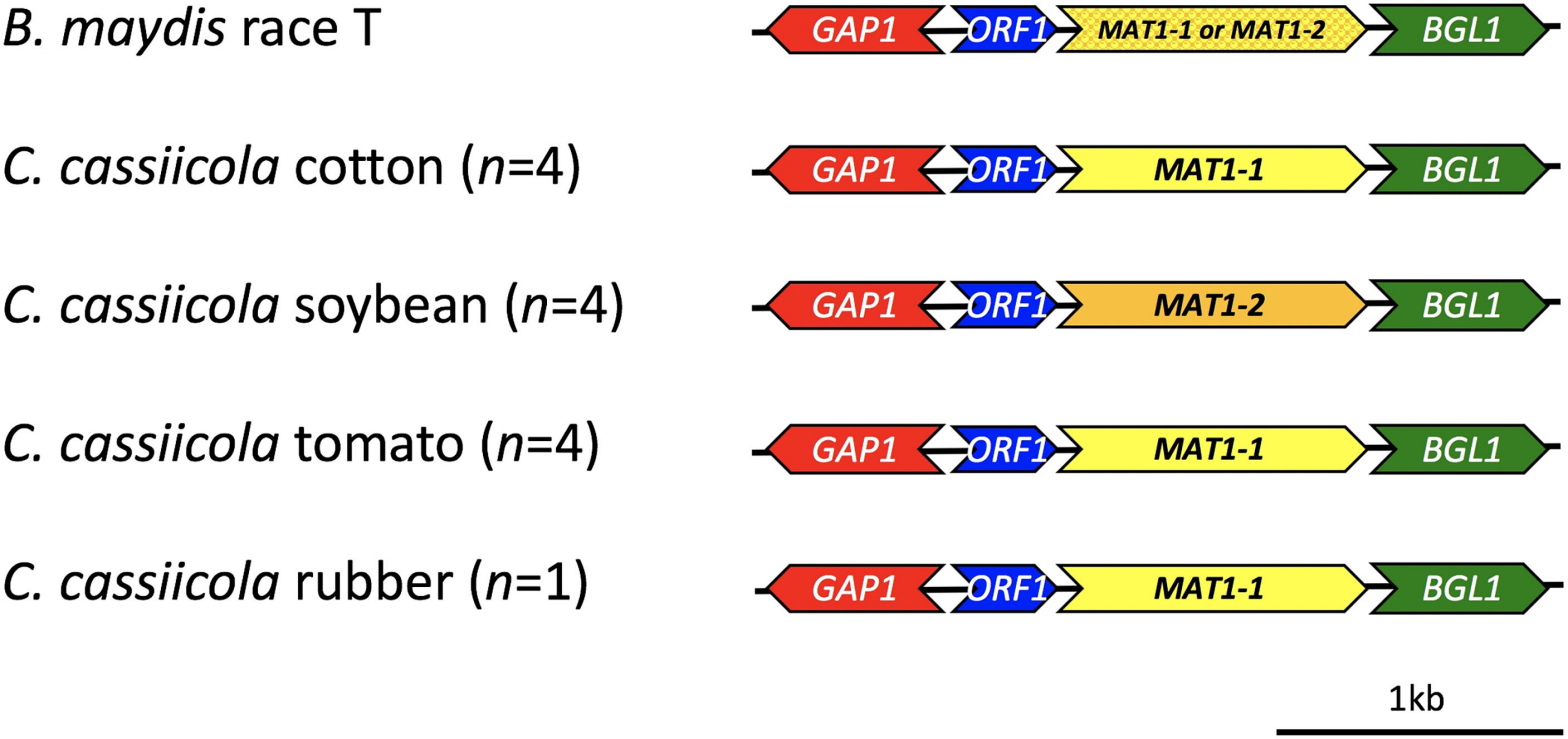
Schematic diagram of the mating-type loci (*MAT1*) in *Corynespora cassiicola* isolates sampled and sequenced for this study. The mating-type idiomorphs *MAT1-1* (AAB82945.1) and *MAT1-2* (AAB84004.1), as well as the flanking genes *gap1* (AAB82943.1), unknown orf (AAB82944.1), and *bgl1* (AAB82946.1) from *Bipolaris maydis* were used as references for comparison.

The mating-type locus was identified in all of the draft *C. cassiicola* genomes, as well as the reference genome (**Figure 4**, **Supplemental Table S1**). The genes flanking the *MAT1* locus in all of the assembled genomes were also identified. The structure of *MAT1-1* in *C. cassiicola* is similar to *B. maydis* (Debuchy and Turgeon, 2006) with isolates having either the *MAT1-1-1* or *MAT1-2-1* gene flanked by *ß-glucosidase* (*BGL1*) downstream of *MAT1* and an unknown open reading frame (*ORF1)* and *GTPase activating protein* (*GAP1)* upstream of *MAT1*. The 8 sequenced isolates of *C. cassiicola* from cotton, tomato, and the reference isolate from rubber have the *MAT1-1* idiomorph, whereas the four from soybean have the *MAT1-2* idiomorph.

### PCR-based marker for *MAT1* in *C. cassiicola*

The primers CCMAT1-1F2 and CCMAT1-1R2 for *MAT1-1* marker and then CCMAT1-2F4 and CCMAT1-2R4 for *MAT1-2* marker (**Table 2**) consistently amplified the corresponding mating-type genes in *C. cassiicola* using a multiplexed reaction. PCR amplification confirmed on gel electrophoresis showed fragment sizes that are 1494 bp and 998 bp for *MAT1-1* and *MAT1-2*, respectively (**Supplemental Figure S1**).

The 25 C*. cassiicola* isolates and the four whole genome sequenced isolates from cotton in the southeastern U.S. all have only *MAT1-1*, the 20 isolates and the four whole genome sequenced isolates from soybean in the southeastern U.S. all have only *MAT1-2*, whereas the 13 isolates from tomato in the southeastern U.S. have either *MAT1-1* (n = 10) or *MAT1-2* (n = 3) (**Supplemental Table S2**). The four genome sequenced isolates from tomato have *MAT1-1.*

## Discussion

In this study, twelve *C. cassiicola* isolates from three host plants (cotton, soybean and tomato) were sequenced with Illumina technology and assembled *de novo*. The assembled genomes of *C. cassiicola* ranged from 45 Mbp to 57 Mbp in genome length (**Table 1**). We hypothesize that the variation in the size can be attributed to intraspecies differences in the phylogenetic lineages (PL). Three of the larger genomes here are from tomato isolates. Previous studies have shown that this lineage (PL4) is quite distinct from the lineage that contains the cotton and soybean isolates (PL1) and could explain the observed differences (Dixon et al., 2009; Sumabat et al., 2018a). The annotated CCP *C. cassiicola* reference genome from rubber is 44.9 Mbp (Lopez et al., 2018) and an average genome size for members of Dothideomycetes is 44.6 Mbp (Mohanta and Bae, 2015), which indicates that the range of our assembled genomes is similar in length to the references. Four of the genomes – CM13, CVa5, Ssta1, and TCl3 – had lower mean sequencing read coverage depths (~50×) relative to the other genomes (~150×). This is evident by the low N50 value indicated by smaller length of the contigs as well as the high L50 value indicative of a higher number of contigs suggesting more fragmented sequence reads. However, the overall genome assemblies were similar, as reflected in the genome sizes as well as in the genome completeness assessment by BUSCO analysis.

Homologs of the cassiicolin-encoding genes were detected in all sequenced cotton and soybean isolates (**Figure 1**). All four sequenced cotton isolates have two *Cas* variants (*Cas1* and *Cas2*); two of the sequenced soybean isolates have only *Cas2*, one has only *Cas6*, and one has both *Cas2* and *Cas6*. Three soybean isolates from Brazil were shown to have only *Cas2* (Déon et al., 2014). Two different *Cas* genes within the same genome has been previously documented (Déon et al., 2014). Two isolates from soybean from Brazil and one isolate from cotton from Brazil were all shown to have both *Cas2* and *Cas6*. Interestingly, population genetic analyses have shown *C. cassiicola* isolates from cotton and soybean sampled from Brazil cluster with the soybean isolates from the southeastern US, whereas the cotton isolates from the southeastern US form a distinct population (Sumabat et al., 2018b). Although there was some variation in the nucleotide sequences of the *Cas* precursor genes, the predicted amino acid sequences of the *Cas1*, *Cas2* and *Cas6* variants identified in the *C. cassiicola* isolates from cotton and soybean from the southeastern US (**Figure 2**) were identical to those of the same previously described *Cas* variants (Déon et al., 2014). Although *Cas* variants were detected *in silico*, it is not clear if these genes are expressed, and if so, what role they have in pathogenicity, host specialization, and virulence of *C. cassiicola* from epidemics in the US. Functional characterization of *Cas1* in an isolate from rubber showed that it is involved in virulence (Ribeiro et al., 2019). Additional functional analyses, such as construction of genetic knockouts, would need to be conducted to evaluate the roles of *Cas* variants in *C. cassiicola* isolates in the southeastern US, and their role(s) as virulence factors or necrotrophic effectors in *C. cassiicola* on cotton and soybean.

Interestingly, no *Cas* variants were detected in any of the assembled genomes of *C. cassiicola* isolates from tomato. Moreover, isolates from tomato were not shown to produce cassiicolin in previous studies (Déon et al., 2014), and the phytotoxin(s) produced by isolates on tomato has yet to be characterized (Onesirosan et al., 1975). In addition, the lineage of *C. cassiicola* to which isolates from tomato in the southeastern US belong is distantly related to the lineage to which the cotton and soybean isolates belong. This evolutionary distance may explain the absence of cassiicolin production in isolates from tomato (Sumabat et al., 2018a; Sumabat et al., 2018b).

A gene cluster containing the orthologs of the *Tox1* genes in *B. maydis* race T has been found in *C. cassiicola* from tomato, cucumber, and rubber (Condon et al., 2018). T-toxin biosynthesis requires at least nine genes that encode for the following *Tox1* genes: polyketide synthases (*pks1* and *pks2*), decarboxylase (*dec1*), dehydrogenases (*lam1*, *oxi1*, *red1*, *red2*, and *red3*), and a hypothetical protein with unknown function (*tox9*) (Inderbitzin et al., 2010). While these genes in *B. maydis* are located on two unlinked loci, the Tox1 orthologs from *C. cassiicola* were located in a compact linear array (Condon et al., 2018). This complete set of nine genes was detected in all of our assembled genomes of *C. cassiicola* from cotton, soybean, and tomato, as well as from rubber (**Figure 3**). It is unlikely that *C. cassiicola* produces T-toxin since a microbial bioassay with *Escherichia coli* cells with the Urf13 protein from T-cms maize were not killed as would be expected if T-toxin were produced (Condon et al., 2018). However, all of the *Tox1-like* genes in *C. cassiicola* were expressed. Although a *Tox1-like* gene cluster was detected in *C. cassiicola*, it is possible that a different compound with a different biological function is synthesized in *C. cassiicola*, or the genes synthesize an entirely different metabolite with a different target that has yet to be characterized.

Additionally, gene clusters with genes showing similarity to other putative secondary metabolite or necrotrophic effector genes were detected *in silico* in the *C. cassiicola* genomes. These included genes involved in synthesis of brefeldin, ectoine, terrein, aspirochlorine, zearalenone, and acetylaranotin. This suggests the possible involvement of additional secondary metabolites in pathogenicity and virulence particularly those from tomato, where none of the *Cas* variants were detected. *In silico* prediction from other recent studies have identified putative effectors in *C. cassiicola* (Looi et al., 2017) as well as those previously detected in other fungal taxa with necrotrophic lifestyle and broad host range (Lopez et al., 2018)

To better understand the reproductive biology of *C. cassiicola*, the mating-type locus (*MAT1*) was identified in all 12 assembled genomes of *C. cassiicola* from cotton, soybean, tomato, and the reference genome CCP from rubber. This locus has not been previously identified despite the fact that *C. cassiicola* has long been reported as strictly asexual or clonal (Dixon et al., 2009; Déon et al., 2014; Sumabat et al., 2018a; Sumabat et al., 2018b). Only one mating-type idiomorph was detected within each of the *C. cassiicola* genomes; however, both mating types were detected among sequenced genomes indicating that *C. cassiicola* is heterothallic. The four isolates we sequenced from cotton and the reference isolate from rubber have only *MAT1-1*, whereas the four isolates we sequenced from soybean have only *MAT1-2*. The four isolates we sequenced from tomato have *MAT1-1*; however, when we used the PCR-based mating-type assay for *C. cassiicola* that we developed here, we detected additional tomato isolates with either *MAT1*-1 or *MAT1-2*. The additional isolates from cotton and soybean were all *MAT1-1* or *MAT1-2*, respectively. Additional studies that identify the mating-type of more isolates from these host plants, as well as from other hosts, especially those that have evolved with a different ecological lifestyle, are needed to determine if host-specialized populations are reproductively isolated since each may contain isolates of only a single mating-type. Moreover, some of these populations and lineages may have diverged due to strictly clonal reproduction. However, the *C. cassiicola* populations from tomato in the southeastern US have both mating types and could potentially reproduce sexually. Determination of the mating-type ratio among a larger set of isolates from tomato or other hosts would help us to infer if cryptic sexual reproduction occurs within or among these populations or lineages (Debuchy and Turgeon, 2006; Brewer et al., 2011); a mating-type ratio not significantly different from 1:1 is expected in randomly mating populations.

The *in silico* identification of putative necrotrophic effectors in *C. cassiicola* does not provide direct evidence for host specialization but allows further investigation on the evolution and function of these different genes in host specialized populations. Further exploration of their evolutionary pathways *via* diverse mechanisms such as horizontal gene transfer, a mechanism for virulence and host specificity that has been documented for several fungal pathogens is now possible. Additionally, we can now target these genes for functional analyses related to host specificity.

## Data Availability Statement

The datasets presented in this study can be found in online repositories. The names of the repository/repositories and accession number(s) can be found below: https://www.ncbi.nlm.nih.gov/, PRJNA549429.

## Author Contributions

LD, RK and MB conceived and designed the research. LD conducted the experiments. LD and MB analyzed the data. LD, RK and MB wrote the manuscript. All authors contributed to the article and approved the submitted version.

## Funding

Funding for this research project was provided by Cotton Incorporated contract 14-280 to MTB

## Conflict of Interest

The authors declare that the research was conducted in the absence of any commercial or financial relationships that could be construed as a potential conflict of interest.

## Publisher’s Note

All claims expressed in this article are solely those of the authors and do not necessarily represent those of their affiliated organizations, or those of the publisher, the editors and the reviewers. Any product that may be evaluated in this article, or claim that may be made by its manufacturer, is not guaranteed or endorsed by the publisher.

## Supplementary Material

Supplementary Figure 1 Gel image of PCR products amplified by the multiplexed PCR-based mating-type assay for *C. cassiicola*. Fragment sizes are 1494 bp for isolates with *MAT1-1* (lane indicated by “1”) and 998 bp for isolates with *MAT1-2* (lane indicated by “2”). “M” indicates the lane with the size marker. Isolate names are listed at the top of each lane.
