# Supplementary figures and images for: Comparative genomics of host-specialized populations of Corynespora cassiicola causing target spot epidemics in the southeastern United States
Source: Front Fungal Biol. 2022 Jul 22;3:910232. doi: 10.3389/ffunb.2022.910232 (PMC10512278; doi:10.3389/ffunb.2022.910232)

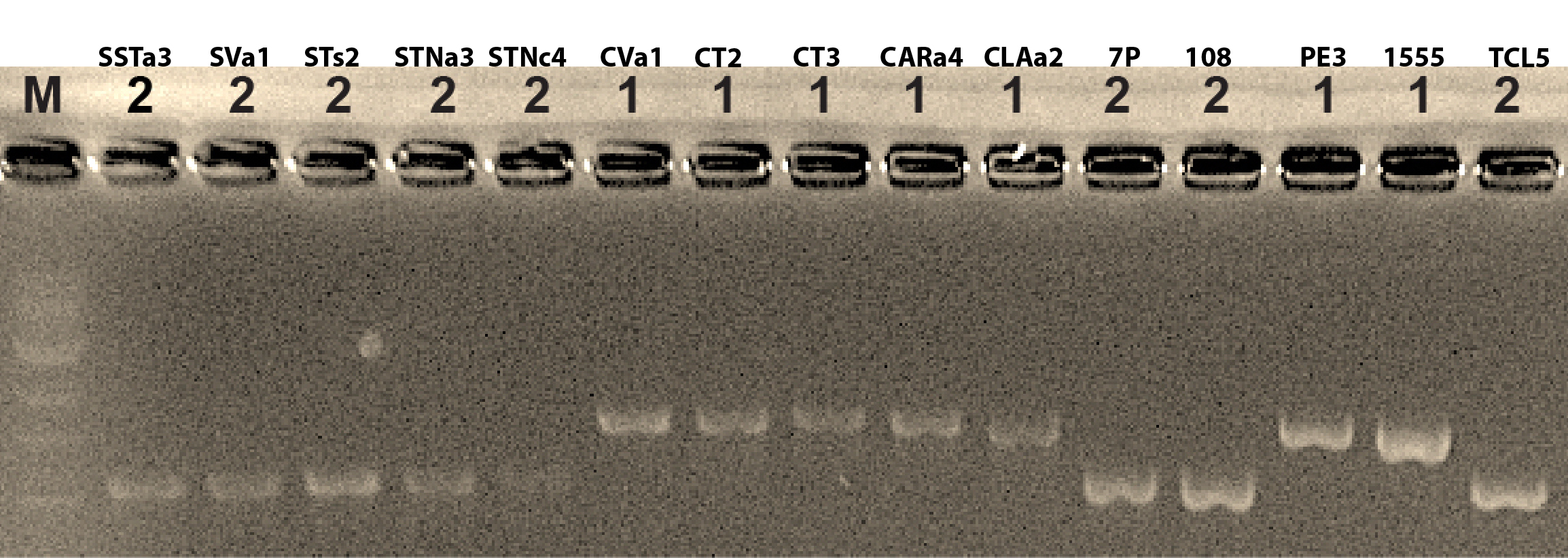

Supplement: Supplementary Figure 1 — Gel image of PCR products amplified by the multiplexed PCR-based mating-type assay for C. cassiicola. Fragment sizes are 1494 bp for isolates with MAT1-1 (lane indicated by “1”) and 998 bp for isolates with MAT1-2 (lane indicated by “2”). “M” indicates the lane with the size marker. Isolate names are listed at the top of each lane. [file Image_1.jpeg]
