# Supplementary material for: Comparative genomics of host-specialized populations of Corynespora cassiicola causing target spot epidemics in the southeastern United States
Source: Front Fungal Biol. 2022 Jul 22;3:910232. doi: 10.3389/ffunb.2022.910232 (PMC10512278; doi:10.3389/ffunb.2022.910232)
Supplement: Supplementary file 2 [file Table_1.docx]

Supplemental Table S1. Location of cassiicolin-encoding genes and mating-type loci in *Corynespora cassiicola* genomes.

| **Isolate** | ***Cas1*** | ***Cas2*** | ***Cas6*** | ***MAT1-1*** | ***MAT1-2*** |
| --- | --- | --- | --- | --- | --- |
| 1. CAL-4 | Contig 484 | Contig 342 | undetected | Contig 29 | undetected |
| 1. CM13 | Contig 452 | Contig 2274 | undetected | Contig 147 | undetected |
| 1. CVa-5 | Contig 698 | Contig 320 | undetected | Contig 87 | undetected |
| 1. FlM4 | Contig 486 | Contig 342 | undetected | Contig 26 | undetected |
| 1. SMR2 | undetected | undetected | Contig 637 | undetected | Contig 16 |
| 1. SAR-9 | undetected | Contig 296 | undetected | undetected | Contig 30 |
| 1. SsTa1 | undetected | Contig 490 | Contig 1147 | undetected | Contig 88 |
| 1. STNa1 | undetected | Contig 311 | undetected | undetected | Contig 22 |
| 1. 1343 | undetected | undetected | undetected | Contig 50 | undetected |
| 1. 1551 | undetected | undetected | undetected | Contig 140 | undetected |
| 1. TCf2 | undetected | undetected | undetected | Contig 144 | undetected |
| 1. TCl3 | undetected | undetected | undetected | Contig 153 | undetected |
