# Supplementary material for: Comparative genomics of host-specialized populations of Corynespora cassiicola causing target spot epidemics in the southeastern United States
Source: Front Fungal Biol. 2022 Jul 22;3:910232. doi: 10.3389/ffunb.2022.910232 (PMC10512278; doi:10.3389/ffunb.2022.910232)
Supplement: Supplementary file 3 [file Table_2.docx]

Supplemental Table S2. Original host, sampling location, isolate name and mating type of *Corynespora cassiicola* isolates screened by PCR.

| Original host | Original location | Isolate name | Mating-type by PCR assay  (*also identified in genome) |
| --- | --- | --- | --- |
| Cotton | Macon Co., AL | CAL-4 | *MAT1-1** |
| Cotton | Duval Co., FL | FlM4 | *MAT1-1** |
| Cotton | Mitchell Co., GA | CM13 | *MAT1-1** |
| Cotton | Suffolk, VA | CVa5 | *MAT1-1** |
| Cotton | Mitchell Co., GA | CM2 | *MAT1-1* |
| Cotton | Mitchell Co., GA | CM3 | *MAT1-1* |
| Cotton | Mitchell Co., GA | CM4 | *MAT1-1* |
| Cotton | Mitchell Co., GA | CM5 | *MAT1-1* |
| Cotton | Headland, AL | CAL1 | *MAT1-1* |
| Cotton | Headland, AL | CAL2 | *MAT1-1* |
| Cotton | Headland, AL | CAL2a | *MAT1-1* |
| Cotton | Suffolk, VA | CVa1 | *MAT1-1* |
| Cotton | Suffolk, VA | CVa2 | *MAT1-1* |
| Cotton | Suffolk, VA | CVa3 | *MAT1-1* |
| Cotton | Tift Co., GA | CT1 | *MAT1-1* |
| Cotton | Tift Co., GA | CT2 | *MAT1-1* |
| Cotton | Tift Co., GA | CT3 | *MAT1-1* |
| Cotton | Dyer Co., TN | CTNa-1 | *MAT1-1* |
| Cotton | Dyer Co., TN | CTNa-2 | *MAT1-1* |
| Cotton | Dyer Co., TN | CTNa-3 | *MAT1-1* |
| Cotton | Thomas Co., GA | TCU1 | *MAT1-1* |
| Cotton | Thomas Co., GA | TCU2 | *MAT1-1* |
| Cotton | Thomas Co., GA | TCU3 | *MAT1-1* |
| Cotton | Jonesboro, AR | CARa-2 | *MAT1-1* |
| Cotton | Jonesboro, AR | CARa-3 | *MAT1-1* |
| Cotton | Jonesboro, AR | CRa-4 | *MAT1-1* |
| Cotton | LA | CLAa-1 | *MAT1-1* |
| Cotton | LA | CLAa-2 | *MAT1-1* |
| Cotton | LA | CLAb-2 | *MAT1-1* |
| Soybean | Poinsett Co., AR | SAR-9 | *MAT1-2** |
| Soybean | Marion Co., GA | SMR2 | *MAT1-2** |
| Soybean | Tift Co., GA | SSTa1 | *MAT1-2** |
| Soybean | Gibson Co., TN | STNa-1 | *MAT1-2** |
| Soybean | Tift Co., GA | SSTa2 | *MAT1-2* |
| Soybean | Tift Co., GA | SSTa3 | *MAT1-2* |
| Soybean | Marion Co., GA | SMR3 | *MAT1-2* |
| Soybean | Suffolk, VA | SVa1 | *MAT1-2* |
| Soybean | Madison Co., TN | STs1 | *MAT1-2* |
| Soybean | Madison Co., TN | STs2 | *MAT1-2* |
| Soybean | Gibson Co., TN | STNa2 | *MAT1-2* |
| Soybean | Gibson Co., TN | STNa3 | *MAT1-2* |
| Soybean | Gibson Co., TN | STNb1 | *MAT1-2* |
| Soybean | Gibson Co., TN | STNb2 | *MAT1-2* |
| Soybean | Gibson Co., TN | STNb3 | *MAT1-2* |
| Soybean | Gibson Co., TN | STNc3 | *MAT1-2* |
| Soybean | Gibson Co., TN | STNc4 | *MAT1-2* |
| Soybean | Gibson Co., TN | STNd3 | *MAT1-2* |
| Soybean | Gibson Co., TN | STNd4 | *MAT1-2* |
| Soybean | Tift Co., GA | SGa2 | *MAT1-2* |
| Soybean | Tift Co., GA | SSTa4 | *MAT1-2* |
| Soybean | Tift Co., GA | SSTa5 | *MAT1-2* |
| Soybean | Poinsett Co., AR | SAR2 | *MAT1-2* |
| Soybean | Poinsett Co., AR | SAR4 | *MAT1-2* |
| Tomato | Hillsborough Co., FL | 1343 | *MAT1-1** |
| Tomato | Hillsborough Co., FL | 1551 | *MAT1-1** |
| Tomato | Cairo, GA | TCl3 | *MAT1-1** |
| Tomato | Cairo, GA | TCf2 | *MAT1-1** |
| Tomato | Hillsborough Co., FL | 1555 | *MAT1-1* |
| Tomato | Hillsborough Co., FL | 7P | *MAT1-2* |
| Tomato | Hillsborough Co., FL | 108 | *MAT1-2* |
| Tomato | Cairo, GA | TCl1 | *MAT1-1* |
| Tomato | Cairo, GA | TCl2 | *MAT1-1* |
| Tomato | Cairo, GA | TCl4 | *MAT1-1* |
| Tomato | Cairo, GA | TCl5 | *MAT1-2* |
| Tomato | Cairo, GA | TCf1 | *MAT1-1* |
| Tomato | Echols Co., GA | PE1 | *MAT1-1* |
| Tomato | Echols Co., GA | PE2 | *MAT1-1* |
| Tomato | Echols Co., GA | PE3 | *MAT1-1* |
| Tomato | Echols Co., GA | PE4 | *MAT1-1* |
| Tomato | Echols Co., GA | PE5 | *MAT1-1* |
